# Hemorrhagic pericardial tamponade in a hemodialysis patient with catheter-related superior vena cava syndrome: a case report

**DOI:** 10.1186/s13019-024-02624-y

**Published:** 2024-03-23

**Authors:** Xiaohong Zhao, Kang Wang

**Affiliations:** 1 Department of Nephrology, Shenzhen People’s Hospital (The Second Clinical Medical College, Jinan University; The First Affiliated Hospital, Southern University of Science and Technology), Shenzhen, 518020 Guangdong China; 2 Shenzhen Key Laboratory of Kidney Diseases, Shenzhen People’s Hospital (The Second Clinical Medical College, Jinan University; The First Affiliated Hospital, Southern University of Science and Technology), Shenzhen, 518055 Guangdong China

**Keywords:** Case report, Iatrogenic endovascular injuries, Superior vena cava syndrome, Percutaneous transluminal angioplasty, Percutaneous venous stenting

## Abstract

**Background:**

Iatrogenic complications of endovascular treatment for central venous stenosis have not yet been reported. Here we present a case of a patient on maintenance hemodialysis who developed catheter-related superior vena cava syndrome and subsequently suffered from hemorrhagic pericardial tamponade after undergoing percutaneous transluminal angioplasty and stenting.

**Case presentation:**

A 72-year-old male patient presented with uremia, and had been receiving maintenance hemodialysis for the past five years. The patient initially presented with dysfunction of the dialysis catheter (a cuffed tunneled double-lumen catheter in the right internal jugular vein). Imaging examination revealed a segmental occlusion of the superior vena cava stretching from the distal end of the dialysis catheter up to right atrium entrance, apparent compensatory dilatation of the azygos vein, and abundant subcutaneous collaterals. The patient underwent percutaneous transluminal balloon dilatation and stenting (covered stent) of the superior vena cava in the Cath Lab. During the procedure, with forceful advancement of the guidewire, it was observed to progress for a distance before a “smoke” appeared, and an outward spillage of contrast agent was visible, which suggested a possible vessel puncture leading into the mediastinum. Unfortunately, postoperative hemorrhagic pericardial tamponade occurred and the patient developed cardiogenic shock. He experienced symptoms included chest tightness and breath shortness with a recorded blood pressure of 84/60mmHg. After draining 600 ml of bloody fluid through pericardiocentesis, the patient’s symptoms alleviated and his condition improved.

**Conclusions:**

The case emphasizes the need for increased attention to iatrogenic endovascular injuries during catheter placement and endovascular treatment, such as causing pericardial hemorrhage leading to cardiac tamponade.

## Background

Superior vena cava syndrome (SVCS) is caused by obstruction of venous return, which can be due to various reasons, such as malignancy or, rarely, endovascular devices. This obstruction leads to clinical symptoms, including swelling of the face, neck, trunk, and upper limbs, coughing, chest pain, respiratory distress, headache, visual symptoms, dizziness, syncope, and more [1]. Pericardial tamponade can also occur due to the recurrence of facial or neck malignancy or sepsis [2, 3]. However, there have been no reports of cardiac tamponade associated with SVCS. The use of central venous catheters is a common iatrogenic cause of SVCS [4]. In hemodialysis patients, central venous stenosis (CVS) not only leads to a series of clinical complaints but also causes dysfunction of the dialysis catheter and jeopardizes the future of suitable vascular access in the affected extremity.

At present, percutaneous intervention using transluminal angioplasty and/or stent placement is the preferred treatment for venous stenosis [5]. However, there has been limited research on the iatrogenic complications of percutaneous transluminal angioplasty (PTA) and stenting for the treatment of SVCS.

## Case presentation

In this case report, we present the case of a 72-year-old Chinese male hemodialysis patient with catheter-related SVCS. The patient’s clinical symptoms improved after undergoing PTA and stenting of the superior vena cava (SVC). However, one week after the procedure, the patient developed hemorrhagic pericardial tamponade. Following pericardiocentesis, the patient was discharged in good condition with a functioning catheter.

A 72-year-old male patient presented with uremia, and had been receiving maintenance hemodialysis for the past five years. Following the diagnosis of end-stage renal disease caused by obstructive nephropathy, a left arteriovenous fistula (AVF) was created in 2014 for dialysis. Unfortunately, the AVF malfunctioned after only 9 months. As a result, a cuffed tunneled double-lumen dialysis catheter was placed in the right internal jugular vein (RIJV) of the patient in the local hospital. For various reasons (the absence of surgical facilities at the local hospital and the short duration of the initial fistula usage), the patient refused a second internal fistula surgery thereafter. The patient has been undergoing regular dialysis treatment for the past 5 years using this central venous catheter (CVC). He had experienced a catheter-related infection once. After antimicrobial therapy, the patient continued using the catheter for dialysis. His medical history included urinary calculi, obstructive nephropathy, hypertension and paroxysmal atrial tachycardia (arrhythmia).

In July 2019, the patient began experiencing dysfunction with his dialysis catheter. During dialysis, there was a noticeable decrease in blood flow (approximately 180–190 ml/min), and repeated intraluminal urokinase administrations (injecting steady doses of 5000 U/ml urokinase to fill the entire catheter lumen for 30 min) at the hemodialysis unit did not provide any improvement. As a result, the patient was transferred to our hospital for further treatment. Upon physical examination, the patient appeared well-oriented with stable vital signs. There was mild swelling in the right upper limb and a few visible dilated collateral veins over the chest wall. A slight catheter prolapse was also observed. Laboratory investigations revealed the following results: hemoglobin: 9.9 g/dL, serum calcium: 2.03 mmol/L, serum phosphorus: 2.32 mmol/L, blood urea nitrogen: 17.53 mmol/L, serum creatinine: 1153 umol/L, prothrombin time (PT): measured value 12.3 s (control value 12.2 s, ± 3 s), activated partial thromboplastin time (APTT): measured value 26.7 s (control value 29.5s, ± 10s), and fibrinogen: 4.32 g/L (2–4 g/L). Preoperative cardiac ultrasound showed no obvious abnormalities. A chest X-ray revealed that the distal ends of the catheter were positioned around the sixth thoracic vertebra (upper part of the SVC), suggesting a mispositioned catheter. Further enhanced computed tomography angiography (CTA) confirmed a clear occlusion of the lower part of the SVC, significant compensatory dilatation of the azygos vein, and the presence of abundant subcutaneous collaterals.

**Fig. 1:**
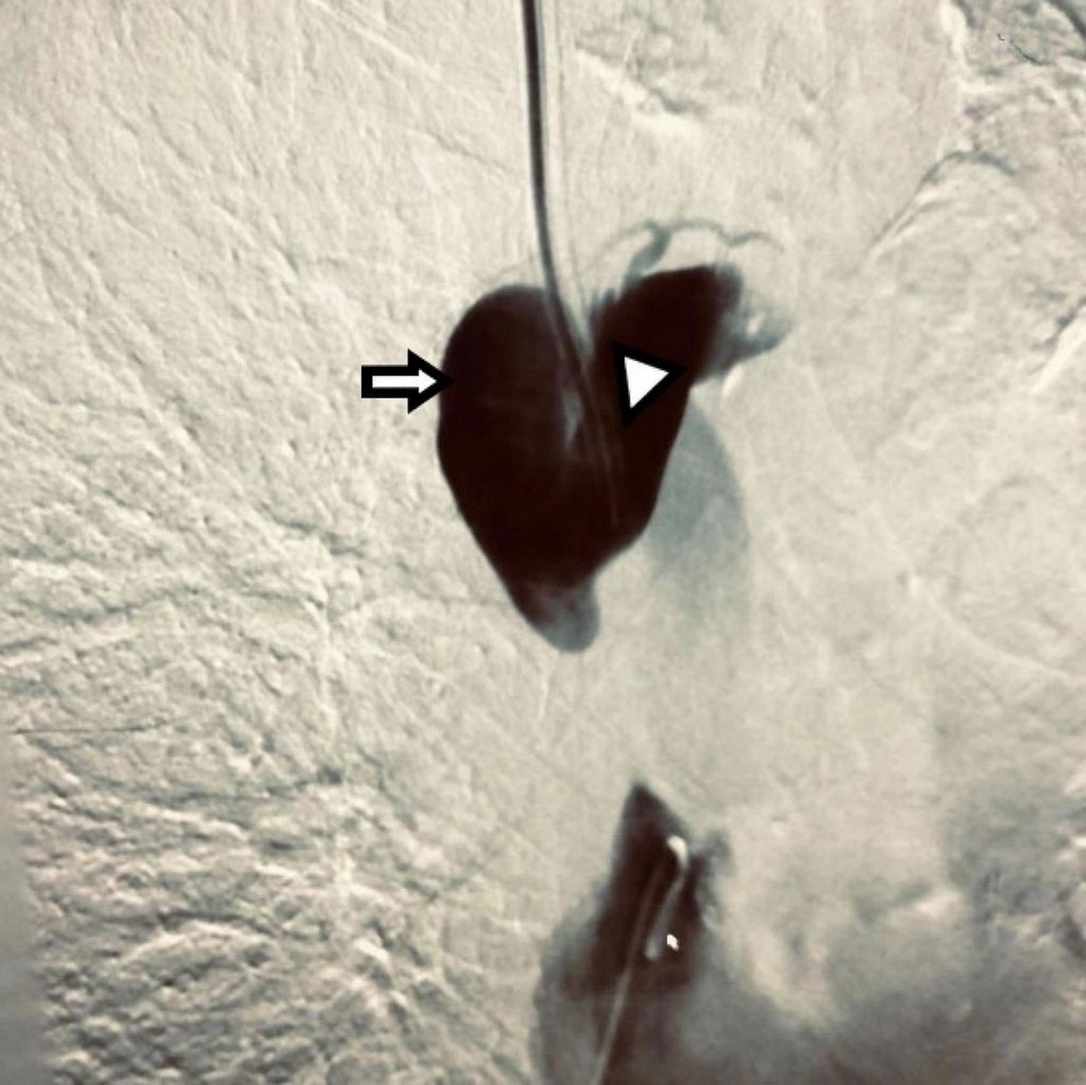
Venography. Venography revealed nearly complete occlusion of the distal end of the catheter in the superior vena cava (SVC). (Arrow: azygos vein; triangle: SVC)

**Fig. 2:**
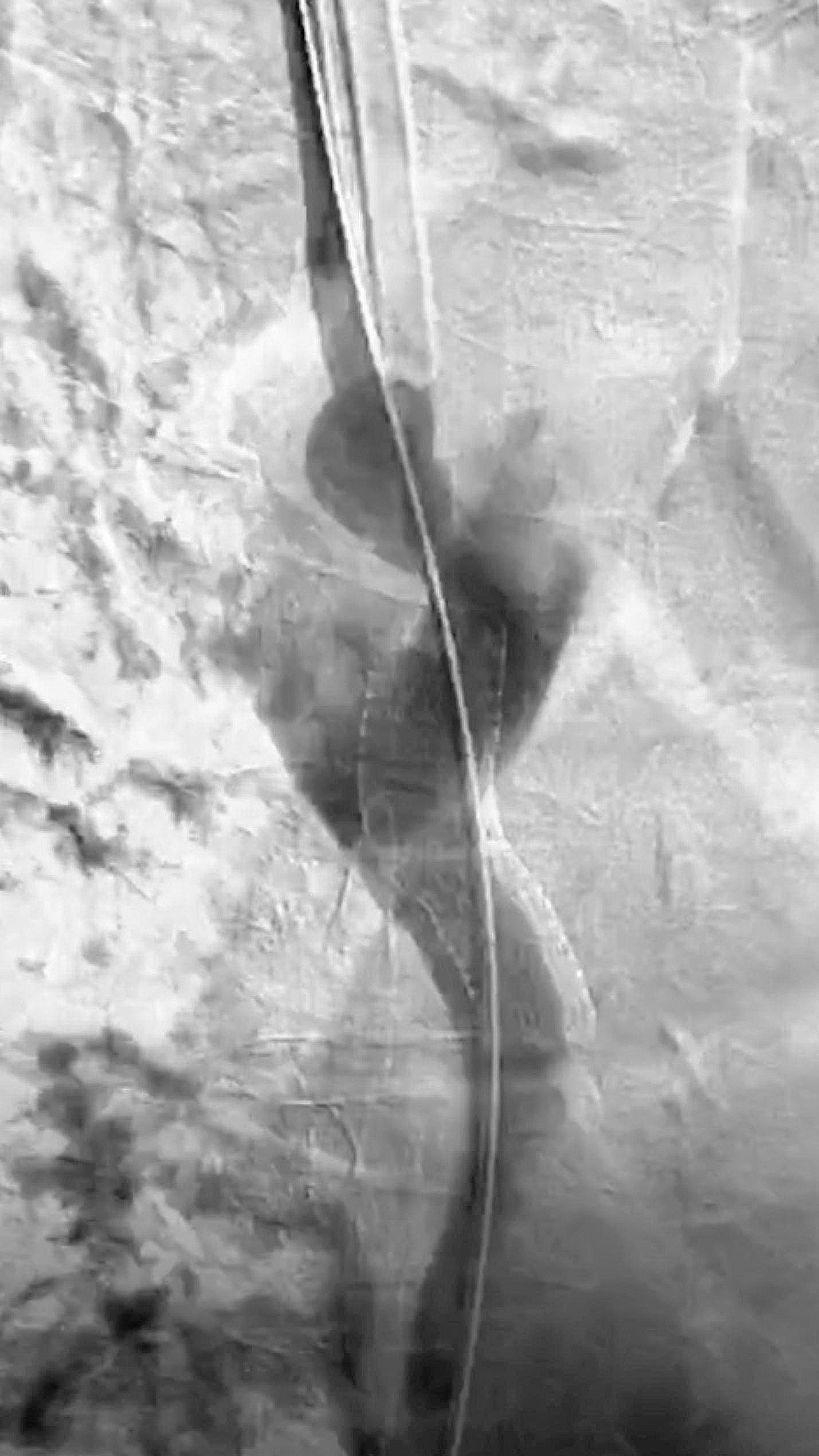
Blood flow restoration after intervention. Venography conducted through the catheter demonstrated restoration of blood flow in the SVC

After diagnosing CVC-related SVCS, a procedure called PTA and endovascular stent placement was performed to treat the stenosis. A catheter was used to carry out a venography, which revealed that the downstream SVC (superior vena cava) from the distal end of the dialysis catheter to the entrance of the right atrium was almost completely blocked, with the occluded segment measuring about 3 cm in length (Fig. 1). During the operation, it was extremely challenging for wire (V-18 Control Wire, Boston Scientific, America, 0.018 in*300 cm) to pass through the severe stenosis. Several unsuccessful attempts were made to clear the occlusion in the SVC. A 6 F sheath dilator was introduced, followed by the insertion of a supporting catheter to hold the opening of the superior vena cava. Later, the V-18 guidewire’s hard tip was used to attempt sharp opening. The advancement of the guidewire met with significant resistance. A “smoking” appearance became noticeable after the guidewire progressed a certain distance, followed by an outward spillage of the contrast agent. Considering that the sharp opening by the guidewire did not enter the lumen of the superior vena cava blood vessel, it may have pierced the vessel and entered the mediastinum. The guidewire was then withdrawn, and the soft tip end of the guidewire was used for further attempts. The patient had no complaints of discomfort and there were no changes in vital signs. The surgery proceeded. During the attempt, the guidewire eventually entered the lumen of the superior vena cava through the right side of the dialysis catheter and confirmed entry into the true lumen of the superior vena cava. Balloon (5 mm) dilation was then performed, followed by the release of a 13 mm*5 cm covered stent (Viabahn, Ameica) to restore blood flow in the SVC (Fig. 2). The follow-up angiography showed that the stent expansion was successful, and the blood flow in the superior vena cava has been fully restored, with no leakage of contrast agent, with no further visualization of peripheral collateral circulation. At the same time, the dialysis catheter was replaced in its original position with both distal ends located inside the right atrium, using the guidewire. No fibrous sheath or thrombosis was found in the original catheter. After the operation, the patient was prescribed anticoagulant therapy with enoxaparin sodium injection (4000 IU, administered via hypodermic injection, once daily). Coagulation tests conducted 4 days post-operation showed the following results: prothrombin time (PT) measured value of 12.8 s (control value 11.9 s, ± 3 s), activated partial thromboplastin time (APTT) measured value of 37.1 s (control value 28.7 s, ± 10 s), fibrinogen level of 5.05 g/L (normal range: 2–4 g/L). The swelling in the patient’s right upper limb was gradually subsiding, and the dilated veins on the chest wall had also diminished. The catheter functioned well, with blood flow increasing to 280 ml/min during dialysis.

**Fig. 3:**
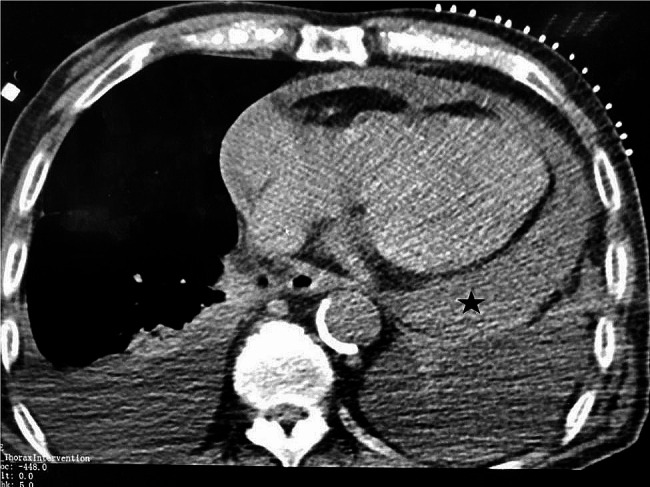
Postoperative chest CT examination. Chest CT examination revealed the presence of pericardial effusion (star)

However, one week after the intervention, the patient began experiencing worsening chest tightness and shortness of breath. Before hemodialysis, the blood pressure was measured at 120/95mmHg and heart rate at 110 beats per minute. However, after 3 h of dialysis with an ultrafiltration of 2200 ml, the patient suddenly developed hypotensive shock with a blood pressure of 84/60mmHg, respiratory rate of 30 breaths per minute, heart rate of 168 beats per minute (showing atrial tachycardia on electrocardiogram), and oxygen saturation of 80% as measured by pulse oximetry. Following rescue efforts, a chest CT scan revealed pericardial effusion (Fig. 3). Approximately 600 ml of bloody fluid was drained through pericardiocentesis, which alleviated the patient’s symptoms of cardiac tamponade. After excluding pericardial effusion caused by tumors or inflammation, we considered the newly developed hemorrhagic pericardial effusion to be related to the endovascular treatment. Eventually, the patient was discharged in good condition with proper functioning of the catheter. The patient has not experienced any recurrence of SVCS, and thus far, the catheter has continued to work well during follow-up visits. We recommended that the patient undergo another venous fistula surgery, but the patient has not agreed so far.

## Discussion and conclusions

The Superior Vena Cava (SVC) is a blood vessel with thin walls that operates under low endovascular pressure. It is elastic and prone to recoil and is formed by the merging of the right and left brachiocephalic veins. When the SVC becomes obstructed, it blocks venous blood return from the upper extremities, head, and neck. However, if the contralateral vein provides adequate compensation, the obstruction may remain asymptomatic. The most common development of collateral venous return to the heart occurs through the azygos/hemiazygos system. In our particular case, the obvious compensatory dilatation of the azygos vein explains why the symptoms of SVC obstruction were not severe. Instead, the patient’s chief complaint was related to catheter dysfunction caused by the blockage.

The most common cause of dysfunction in dialysis catheters is thrombosis of the catheter lumens [5]. If intra-catheter urokinase treatment fails, it suggests other potential problems, such as external fibrin catheter sheath, mispositioned catheter tip, or vascular abnormality. To identify the underlying issues, it is helpful to conduct catheter imaging with contrast infusion. In this patient, the imaging examination revealed that the distal ends of the dialysis catheter were located in the upper part of the SVC. The mispositioned catheter tip and subsequent turbulent flow led to downstream SVC stenosis, resulting in catheter dysfunction. Currently, the favored approach is to place both lumens of the long term RIJV catheter within the right atrium, allowing for high flow during dialysis and reducing the impact of turbulence flow on the blood vessel wall. Both high vascular wall shear stress and vascular compression or distortion play a key role in CVS [4]. Clinicians should pay more attention to iatrogenic SVCS resulting from the use of central venous catheters. First, strict limitations should be placed on indications for their use. The most desirable strategy is the prevention of CVS. Percutaneous intervention with transluminal angioplasty and/or stent placement is the preferred approach for CVS according to the guidelines of the Kidney Disease Outcomes Quality Initiative (K/DOQI) [6]. However, little attention is currently being paid to the complications of endovascular treatment. Despite there being quite a few studies on SVCS, iatrogenic endovascular injuries associated with PTA and stenting treatment have not been noticed. Iatrogenic pericardial effusion and tamponade have been reported as complications associated with a wide variety of structural heart diseases and electrophysiologic interventions [3]. Previous studies have rarely reported on the serious complications of PTA and stenting of the SVC.

Unfortunately, our patient experienced hemorrhagic pericardial tamponade after the intervention. Upon reflection, we have identified several potential causes. First, due to the severity of the occlusion, the guidewire had to be attempted repeatedly, resulting in mechanical injury while passing through the stenosis. Due to the extremely thin guidewire, it was not easy to detect the puncture injury to blood vessels and tissues during the procedure. Second, the subsequent hemorrhage at the site of injury was exacerbated by active anticoagulation therapy following the operation. Last, calcification and sclerosis of the vascular wall in a hemodialysis patient may have also contributed to the complication.

To be honest, due to a lack of relevant experience, we did not consider the possibility of hemorrhage until the patient developed symptoms of pericardial tamponade. This case serves as a reminder that preoperative risk assessment must be conducted with caution before performing PTA and stenting. It is crucial to closely monitor for bleeding and consider reducing the dosage of anticoagulants post-operation if necessary. Furthermore, future improvements in equipment and angioplasty techniques are needed to minimize endovascular injuries and postoperative complications.

The patient’s current condition is satisfactory, with the catheter functioning effectively. However, vigilance is needed to monitor for thrombosis within the stent and the potential for recurrent cardiovascular events in future follow-up visits. Additionally, the complexity of creating an arteriovenous fistula (AVF) in the upper limb under the present circumstances should be acknowledged. If possible, renal replacement methods, other than hemodialysis should be considered.

In conclusion, iatrogenic endovascular injuries during catheter placement and endovascular interventions should be given greater attention in future clinical practice due to the potential for serious complications. Thorough pre-operative evaluation and stringent post-operative observation are crucial. Furthermore, it is important to be alert to mechanical damage, and also consider the possibility of rare complications such as pericardial tamponade.

## Data Availability

Data sharing is not applicable to this article as no datasets were generated or analyzed during the current study.

## Abbreviations

AVF: Arteriovenous fistula
PT: Prothrombin time
APTT: Activated partial thromboplastin time
CTA: Computed tomography angiographic
CVC: Central venous catheter
CVS: Central venous stenosis
K/DOQI: Kidney Disease Outcomes Quality Initiative
PTA: Percutaneous transluminal angioplasty
RIJV: Right internal jugular vein
SVC: Superior vena cava
SVCS: Superior vena cava syndrome
